# Natural Killer Cells in Afferent Lymph Express an Activated Phenotype and Readily Produce IFN-γ

**DOI:** 10.3389/fimmu.2013.00395

**Published:** 2013-11-22

**Authors:** Hege Lund, Preben Boysen, Jayne C. Hope, Siri K. Sjurseth, Anne K. Storset

**Affiliations:** ^1^Department of Food Safety and Infection Biology, Norwegian School of Veterinary Science, Oslo, Norway; ^2^The Roslin Institute, The University of Edinburgh, Edinburgh, UK; ^3^Department of Immunology, Norwegian Veterinary Institute, Oslo, Norway

**Keywords:** natural killer cells, afferent lymph, bovine, trafficking, activation, interferon-γ

## Abstract

Natural killer (NK) cells are motile cells that migrate between peripheral blood (PB), lymph nodes (LNs), and various organs. Domestic animals have frequently been used to study cellular migration, and offer unique opportunities for such studies. The aim of this study was to characterize the phenotype and cytokine producing capacity of NK cells in bovine skin-draining lymph. NKp46/NCR1^+^ CD3^−^ cells constituted 2–11% of mononuclear cells in afferent lymph (AL), a majority of cells were CD16^+^, CD8α^+^, and CD2^−/low^, and elevated CD25 and CD44 expression indicated an activated phenotype. Interestingly, significantly fewer AL NK cells expressed the early activation marker CD69 compared to PB NK cells. A large proportion of lymph and blood NK cells produced interferon (IFN)-γ following stimulation with IL-2 and IL-12. Notably, in AL, but not blood, a similar amount of IFN-γ^+^ NK cells was observed when cells were stimulated with IL-12 alone. Overall, AL NK cells were more similar to LN-residing NK cells than those circulating in PB. We conclude that AL appears to be an important migration route for tissue-activated NK cells, and may represent an alternative route for NK cell traffic to LNs. These findings may have important implications in the development of adjuvant strategies that aim to target NK cells in a vaccine response.

## Introduction

Natural killer (NK) cells are innate lymphocytes that act as early responders during infection or inflammation by means of cytotoxicity and production of immunoregulatory cytokines. Although NK cells are found widely distributed in non-lymphoid and lymphoid tissues in human, mice, swine, and cattle (1–4), much is still unknown regarding their recirculation and the chemokines and homing molecules controlling these movements (5, 6).

During infection or inflammation, murine NK cells gain increased entry from peripheral blood (PB) into lymph nodes (LNs) via high-endothelial venules (HEVs). Interaction with activated dendritic cells (DCs) in the LN is believed to be important for the priming of NK cells (7), upon which NK cells express the early activation marker CD69 (8), but additional signals are needed for NK cells to reach full activation and mediate effector functions such as cytotoxicity and interferon (IFN)-γ production (9). NK cells are believed to provide an early source of IFN-γ required for a Th1 polarization of the immune response *in vivo* in mice (10, 11). These events may be essential for the promotion of efficient Th1 targeting of vaccines (12). Since many vaccines are delivered to the skin (intradermal or subcutaneous) understanding the function and phenotype of cells draining from this site can provide important mechanistic insights to assist effective vaccine design and delivery. Whilst a number of studies have focused on DC in this context little is currently understood about other innate cells draining from the periphery.

The migration of NK cells from LNs via the efferent lymph, into the PB and subsequent entry into inflamed tissue has been demonstrated (2), but little is known of NK cell trafficking after entry into tissues. Afferent lymphatics drain T cells and DCs from tissues (13). The presence of NK cells has been reported in afferent lymph (AL) draining the skin in humans (14, 15) and domestic animals (16–18), but these cells have never been further characterized. In human, AL was accessed by direct microsurgical cannulation of a superficial lymphatic vessel (19, 20), giving access to cell populations originating from healthy normal skin (15, 21) and allergic contact dermatitis affected skin (14). CD56^+^ cells were observed in human AL at a significantly lower percentage than in PB (14, 15). A recent paper describes the presence of NKp46/NCR1^+^ cells in seroma fluid believed to be an accumulation of AL (22). To the authors’ knowledge, the three latter studies provide the only observations of NK cells in human AL to date.

The method of pseudo-afferent lymphatic vessel cannulation has been established in various animal models, including sheep (23–25), cattle (17, 26), swine (27), and rat (28), and provides a unique model that has generated a large body of our general knowledge of lymphatic cellular migration from peripheral tissues to the draining LN (23). The application of this model in cattle enables the study of NK cells, which have been relatively well characterized in this species (29, 30). Bovine NK cells, defined as NKp46/NCR1^+^ CD3^−^ lymphocytes, can be divided into a CD2^+^ subset dominating in PB and a CD2^−/low^ subset dominating in LNs (31). The latter subset carry higher levels of CD16, CD8α, and the activation markers CD25 and CD44, are particularly strong IFN-γ producers, and dominate following *in vitro* stimulation with IL-2 or IL-15 (32). Although the bovine subsets are not directly comparable to humans, bovine CD2^−/low^ NK cells to a large extent resemble the CD56^bright^ phenotype that dominates in human lymphoid tissues in terms of function and distribution (33, 34).

We here examined the phenotype and cytokine producing capacity of NK cells in skin-draining AL under homeostatic conditions, using a bovine cannulation model. AL NK cells were found in a highly activated state and of a similar phenotype as LN-residing NK cells, indicating that these cells may home to the LN. More knowledge of the functional capacity and trafficking pattern of NK cells in AL may help to illuminate central unanswered questions about NK cell recirculation and their role in vaccine responses.

## Materials and Methods

### Animals

Animal experiments were carried out according to guidelines of the UK Home Office and the Norwegian Animal Research Authorities, with full ethics approval.

PB was taken from Norwegian Red (NR) dairy calves (Bos taurus) of both sexes and 6–8 months of age, and collected in EDTA-containing tubes. Animals were clinically healthy cattle from a commercial Norwegian dairy farm. PB from Holstein–Friesian calves at the Institute for Animal Health (IAH) was collected by jugular venepuncture into sodium heparin (Leo Pharma, UK).

Pseudo-AL vessel cannulations at IAH were carried out on conventionally reared British Holstein–Friesian male calves. All animals at IAH were aged between 6 months and 1 year. Paired samples of PB and AL were obtained from one individual NR male calf of 6–8 months of age. Repeated phenotyping and intracellular IFN-γ analysis was performed on material collected from this individual at 2–4 weeks after cannulation, and representative results are included in this study.

### Surgery and afferent lymph collection

Pseudo-AL vessels were generated by surgical removal of superficial cervical LNs and cannulations were performed essentially as previously described (17). Briefly, approximately 8 weeks post-lymphectomy, pseudo-AL vessels were surgically cannulated with sterile, pre-siliconized, and heparinized portex tubing (Portex Ltd.). Catheters were fixed in position, passed externally via a skin incision and adequate flow of lymph was ensured. Lymph was collected at various time points from day 3 to 28 post-surgery into sterile plastic bottles containing heparin (10 U/ml), penicillin (60 μg/ml), and streptomycin (100 μg/ml) (Gibco/Invitrogen), and bottles were replaced every 8–12 h. Animals were injected subcutaneously twice daily with heparin (0.5 ml, 2500 IU, GP Pharmaceuticals) into a site draining to the catheterized lymph vessel. The lymph collected was centrifuged (300 × *g*, 8 min), and AL cells were either used immediately in phenotypic or functional studies or resuspended in FBS Gold (PAA, Pasching, Austria) and 10% DMSO for storage in liquid nitrogen. Bovine PBMC were isolated from EDTA or heparinized blood by density gradient centrifugation (2210 × *g*, 30 min) on lymphoprep (Axis-Shield), and used immediately in phenotypic or functional studies.

### Flow cytometry

Three-color flow cytometric (FCM) analysis of surface markers or intracellular proteins was performed on isolated PBMC or on fresh or previously frozen AL cells. Cells were first stained with LIVE/DEAD^®^ fixable far red dead cell stain for 633 excitation (Invitrogen), following the manufacturer’s instructions. Subsequently, cells were surface labeled with in house produced primary monoclonal antibodies (mAbs) against bovine NKp46/NCR1 [AKS1, IgG1 or AKS6, IgG2b; Ref. (32)], alone or in combination with mouse anti-bovine mAb against one of the following molecules: CD3 (MM1A, IgG1), CD2 (MUC2A, IgG2a), CD8α (BAQ111a, IgM), CD25 (CACT108A, IgG2a), CD44 (BAG40a, IgG3), CD62L (BAQ92A, IgG1), CD69 (KTSN7A, IgG1) (all Monoclonal Antibody Center, Washington State University, Pullman, WA, USA), or mouse anti-human CD16 (KD1, IgG2a) (a kind gift from Daniela Pende, ISTGE, Italy) or PE anti-human CCR7 (3D12, rat IgG2a; BD Biosciences, USA). Secondary antibodies used were polyclonal goat anti-mouse and were either PE-conjugated (Southern Biotech, Birmingham, AL, USA) or Alexa Fluor 488-conjugated (Invitrogen, Eugene, OR, USA). Cells surface labeled with AKS1 were permeabilized and fixed (Cytofix/Cytoperm; BD Biosciences), and further incubated with mouse anti-human perforin (delta g9, IgG2b; BD Biosciences), followed by a PE-conjugated secondary antibody. The method for intracellular staining has been described in detail elsewhere (32). Gating was based on stainings with secondary antibodies only or on non-stimulated controls. Flowcytometry was performed with a FACS Calibur flow cytometer and the CellQuest Pro software (BD Biosciences), and expression was measured as % positive NK cells for bimodal distributions and as mean fluorescence intensity (MFI) for other distributions.

### Intracellular IFN-γ analysis

For the detection of intracellular IFN-γ in NK cells, PBMC and AL cells were added to 24-well plates at a concentration of 10^6^ cells/well in 1 ml RPMI (Gibco/Invitrogen), with added penicillin, streptomycin, and 10% FBS. Cells were incubated at 37°C and with 5% CO_2_ for 24 h in medium only, or in the presence of rbIL-2 (100 U/ml), rhIL-12 (400 pg/ml, eBioscience) or a combination of the two cytokines, or in the presence of rhIL-15 (10 ng/ml, eBioscience) alone or in combination with rhIL-12. Brefeldin A (10 μg/ml, Sigma) was added to cells for the final 4 h of stimulation. Cells were stained with LIVE/DEAD^®^ fixable far red dead cell stain, followed by surface staining against NKp46/NCR1 (AKS6) and a secondary PE-conjugated Ab. Permeabilized and fixed cells were incubated with anti-bovine IFN-γ mAb (clone 6, 19, IgG2a) (a kind gift from Gregers Jungersen at the Technical University of Denmark) and secondary Alexa 488-conjugated antibody. Cells were analyzed by FCM on a FACS Calibur.

### Statistics

Differences between the groups were assessed by the non-parametric Wilcoxon rank-sum test.

## Results

### Phenotype of *ex vivo* NK cells

**Figure 1:**
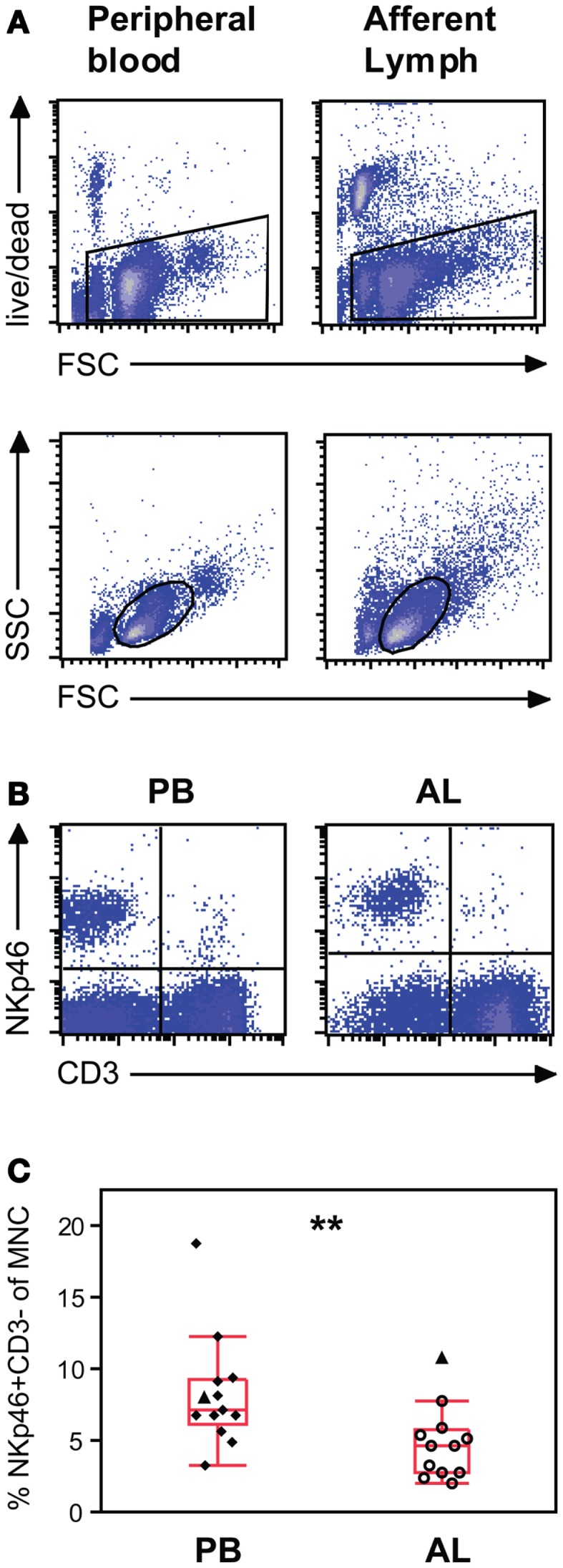
**Natural killer (NK) cell distribution in peripheral blood and afferent lymph from calves *ex vivo***. **(A)** Gating strategy illustrated by representative density plots. Peripheral blood (PB) mononuclear and afferent lymph (AL) cells were gated on viable cells in a FSC/LIVE/DEAD plot (upper panels) and further gated on mononuclear cells in a FSC/SSC plot (lower panels). **(B)** Representative density plots showing NKp46^+^/CD3^−^ NK cells in the upper left quartile and **(C)** distribution of the results showing NK cells as a percentage of mononuclear cells in PB (*n* = 13) and AL (*n* = 12). Quadrants were based on control staining with secondary antibodies alone. Symbols represent individual animals, where open symbols are Holstein–Friesian and filled symbols are Norwegian Red. Filled triangles identify one paired NR individual. Box plots show median and quartiles, and whiskers indicate data points within the 1.5* (interquartile range). Statistically significant differences between groups indicated as **p < 0.01.

By using a model of bovine pseudo-AL vessel cannulation, we examined the NK cell number and phenotype in skin-draining lymph during the steady state. PBMC and AL cells were gated on viable cells in a FSC/LIVE/DEAD plot and further gated on mononuclear cells in a FSC/SSC plot (Figure 1A). Bovine NK cells were defined as NKp46/NCR1^+^ CD3^−^ cells (Figure 1B). NK cells constituted 2–11% of mononuclear cells in AL, with a median value of 4.6% (Figure 1C). In PBMC NK cells were present at 3–18%, with a median value of 7.1%. These results show that PB was significantly more NK cell rich than AL in cattle (*p* < 0.01).

**Figure 2:**
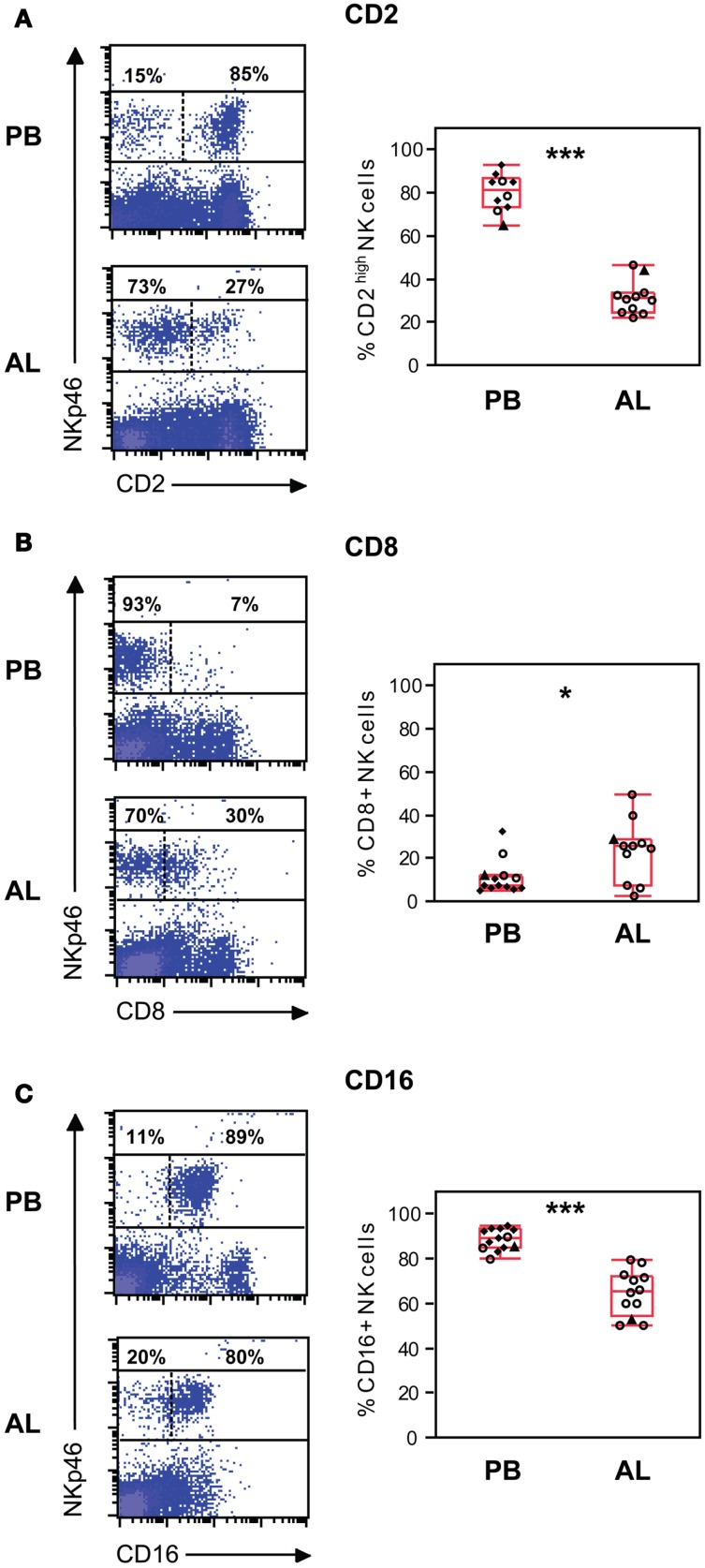
**Phenotype of NK cells *ex vivo***. NK cell expression of the surface molecules CD2 **(A)**, CD8α **(B)**, and CD16 **(C)**. Gating of cells as in Figure 1. Density plots (left panels) of representative animals from peripheral blood (PB) and afferent lymph (AL). Numbers indicate the percentage of NKp46^+^ cells above or below an expression threshold (dashed line) for the indicated molecule. Distribution of the results (right panels) showing % positive NK cells in PB (*n* = 10–13) and AL (*n* = 11–12). Symbols and statistics as in Figure 1 (*p < 0.05, ***p < 0.001).

Viable mononuclear cells from PBMC and AL were further analyzed by FCM for the expression of NKp46/NCR1 in combination with other surface molecules. In AL, the majority of NK cells were CD2^−/low^ while around one third were CD2^high^ (Figure 2A). In contrast, a significantly higher proportion of the CD2^high^ NK cell population was found in PB (*p* < 0.01). Significantly more (*p* < 0.05) NK cells from AL were CD8α^+^ compared to NK cells from PB, although percentages of positive NK cells in AL were highly variable between individuals (Figure 2B). The majority of NK cells in AL and PB expressed CD16 (Figure 2C); however there were significantly more CD16^+^ NK cells present within PB compared to lymph (*p* < 0.001).

**Figure 3:**
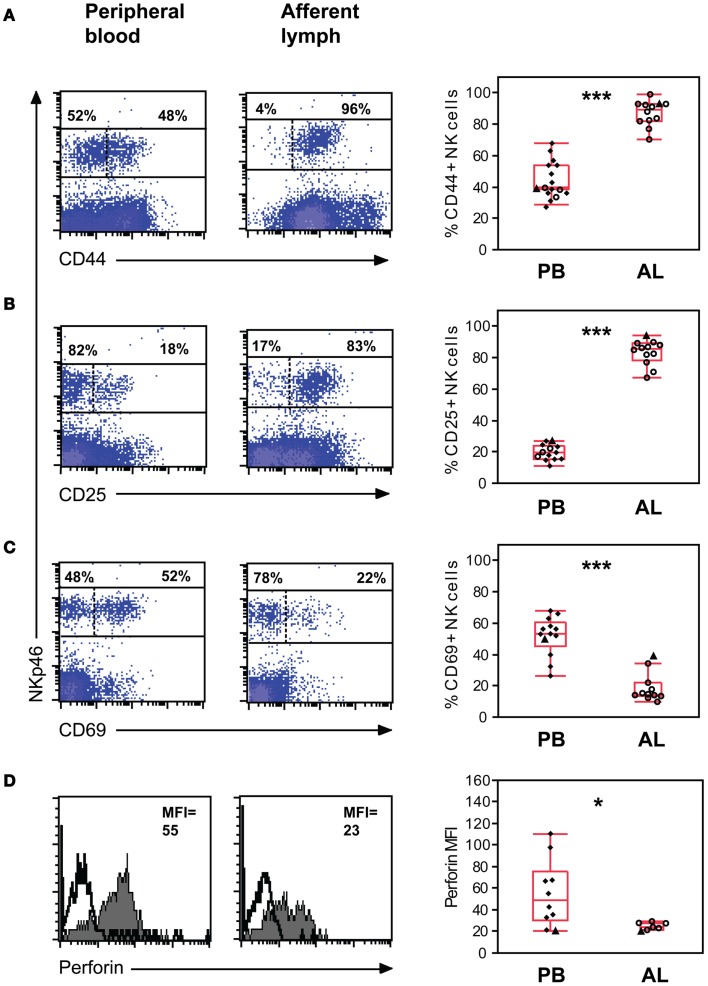
**Expression of activation molecules and intracellular perforin in NK cells *ex vivo***. Expression of the activation molecules CD44 **(A)**, CD25 **(B)**, and CD69 **(C)** on NK cells. Gating of cells as in Figure 1. Density plots of representative animals from peripheral blood (PB, left panels) and afferent lymph (AL, middle panels). Numbers indicate the percentage of NKp46^+^ cells above or below an expression threshold (dashed line) for the indicated molecule. Distribution of the results (right panels) showing % positive NK cells in PB (*n* = 10–13) and AL (*n* = 11–12). **(D)** Intracellular perforin in NK cells, calculated as mean fluorescence intensity (MFI) of the NK cell population. Histograms displaying representative animals from PB (left panel) and AL (middle panel). Filled histograms indicate perforin expression and solid histograms indicate the secondary control staining. Distribution of the results (right panel) showing MFI of NK cells in PB (*n* = 10) and AL (*n* = 7). Symbols and statistics as in Figure 1 (*p < 0.05, ***p < 0.001).

The expression of activation molecules CD44, CD25, and CD69 on viable NK cells from the mononuclear cell fraction was analyzed by FCM. The vast majority of NK cells in AL were CD44^bright^ with no NK cells being completely negative. By contrast in PB significantly fewer NK cells were CD44^+^ (median 40%, *p* < 0.001), with the remaining population being CD44 dim to negative (Figure 3A). Striking differences in CD25 expression were observed between the two compartments. A major CD25^+^ NK population was present in AL, while significantly fewer (*p* < 0.001) CD25^+^ NK cells were present in PB where less than a third of the NK cells expressed this molecule (Figure 3B). In AL, 14% (10–40%) of cattle NK cells expressed the early activation marker CD69, whereas in PB the median percentage of CD69 positive NK cells was found to be significantly higher (*p* < 0.001) at 53% (26–68%) (Figure 3C).

To determine whether skin-draining NK cells are equipped with molecules that allow for LN recruitment, we assessed expression of L-selectin (CD62L) and CCR7. CD62L was expressed on 48% (24–73%) of AL and 59% (39–67%) of PB NK cells, and differences between the groups were not significant (not shown). CCR7 expression was not detected on bovine NK cells in PB or AL (not shown).

Without prior stimulation, AL NK cells expressed intracellular perforin with a median MFI value of 24 (21–28), whereas in PB the median MFI value was found to be significantly higher at 49 (20–110, *p* < 0.05) (Figure 3D).

### Intracellular IFN-γ production of NK cells *in vitro*

**Figure 4:**
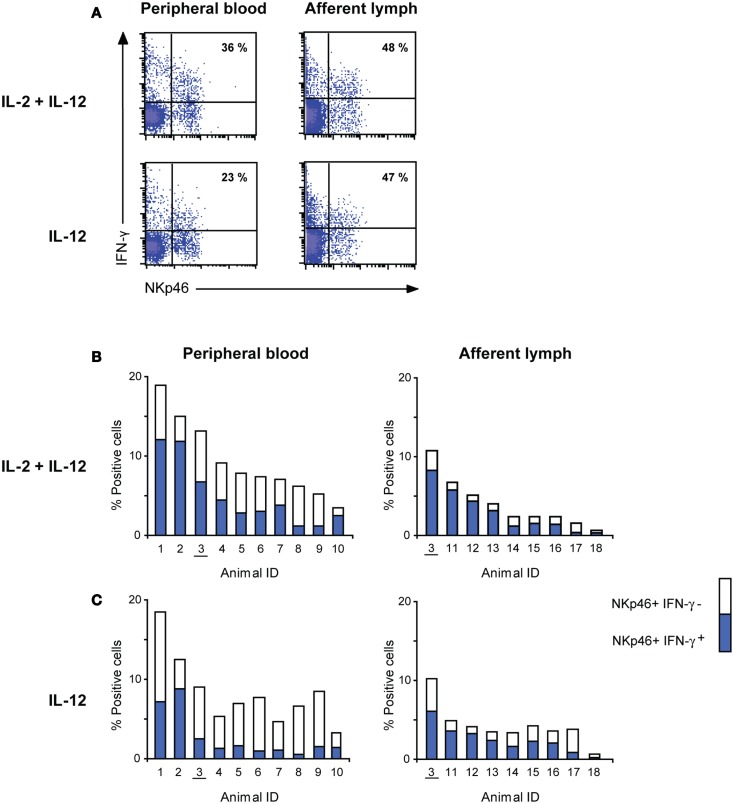
**Interferon-γ producing capacity of NK cells from peripheral blood and afferent lymph**. **(A)** Flow cytometric analysis of intracellular IFN-γ after 24 h of *in vitro* cytokine stimulation. Gating of cells as in Figure 1. Density plots of representative animals from peripheral blood (left panels) and afferent lymph (right panels). Numbers indicate IFN-γ^+^ NK cells (upper right quartile) as percentage of all NK cells. **(B,C)** IFN-γ production of NK cells following cytokine stimulation as in A with IL-2 and IL-12 or only IL-12. Complete columns represent the total percentage of NKp46^+^ cells in the mononuclear cell fraction, while the inner blue columns indicate the percentage of NKp46^+^ IFN-γ^+^ cells in the same fraction. All individual animals are presented with a unique ID, where numbers 1–10 are Norwegian Red and 11–18 are Holstein–Friesian. One paired NRF calf is underscored.

To determine whether NK cells in AL were capable of producing the effector cytokine IFN-γ, we stimulated PBMC and AL cells *in vitro* with cytokines and stained for intracellular IFN-γ after 24 h. Viable mononuclear cells were analyzed by FCM. A large proportion of PB (20–71%) and AL (32–86%) NK cells produced IFN-γ following stimulation with IL-2 and IL-12 (Figure 4A). Similar results were obtained when cells were stimulated with IL-15 and IL-12 (not shown). In AL, a similar amount of IFN-γ^+^ NK cells were observed upon stimulation with IL-12 *only* (24–81%), whereas PB NK cells produced significantly less IFN-γ under this stimulatory condition (9–52%, *p* < 0.05). This difference is illustrated in Figures 4B,C which show the percentage of NKp46/NCR1^+^ IFN-γ^+^ cells in the mononuclear cell fraction in all individuals (Figure 4B). IL-2 or IL-15 alone induced only marginally more IFN-γ^+^ NK cells compared to non-stimulated cells in PB. AL NK cells, however, produced higher amounts of IFN-γ compared to non-stimulated cells when stimulated with IL-2 or IL-15 alone, although at a non-significant level (data not shown).

## Discussion

Although NK cells show a wide tissue distribution (2, 4, 35), the mechanisms by which NK cells traffic through tissues at steady state and following infection are not well characterized. In this study we report the presence and phenotype of NK cells in skin-derived AL from healthy cattle. Our results indicate an alternative route of NK cell recruitment to LNs under physiological conditions, not only from PB via HEV (10, 11), but also from the AL.

Natural killer cells in AL showed a more activated (CD25^+^ and CD44^+^) phenotype than NK cells in PB, and readily produced IFN-γ upon *in vitro* stimulation, raising the question of where and how these cells have been stimulated. The presence of CD56^+^ CD3^−^ NK cells have been reported in human healthy (36, 37) and lesional skin (38), and NK cells were observed in close contact with DCs *in vivo* (36). A close cellular interaction of NK cells and DCs or IL-2 producing T cells in tissues may possibly lead to a further activation of NK cells recruited from PB. It cannot be totally excluded that the activation observed in the current study could be caused by inflammatory stimuli due to the invasive technique used, but cells were only included after stabilization in the cellular composition and animals were carefully monitored for the absence of clinical signs of inflammation; conditions were in accordance to standardized protocols for this technique (17, 24). Thus, the CD2^−/low^ CD25^+^ CD44^+^ phenotype of NK cells in bovine AL, and the resemblance of these with LN-residing NK cells, suggest that under non-inflammatory conditions, NK cells are activated in the tissues, migrate through AL and enter the LNs, where they constitute a large proportion of the residing NK cells.

Natural killer cells in the AL had a significantly lower expression of the early activation marker CD69 than NK in PB, despite the presence of CD25 and potent IFN-γ production which indicate that these cells are not naïve. They may represent a late phase of activation, since *in vitro*, bovine NK cells express high levels of CD69 after 6–12 h of stimulation, followed by a down-regulation in later stages (32). It should also be noted that in T- and B-cells in other species, CD69 is tightly linked to sphingosine-1-phosphate receptor 1 (S1PR_1_) (39). The usage of S1P-receptors in bovine lymphocytes is not known and was not studied here due to limitation of reagents for cattle. However, the observed expression pattern of CD69 would be consistent with reports that this receptor is internalized together with S1PR_1_ in the S1P-rich lymphatic vessels (13), followed by CD69 up-regulation in the S1P-free environment in LNs, where CD69 inhibits S1PR_1_-mediated egress (40, 41). While the mechanisms behind entry of NK cells from AL to LNs remain to be studied, NK cell egress has been shown dependent on S1PR_5_ rather than S1PR_1_ in the mouse, in a process apparently resistant to CD69 inhibition (42, 43). In line with this, we here and previously (32) observed that CD69 is present on a substantial proportion of cattle PB NK cells, although the migration history of these CD69^+^ PB NK cells needs to be clarified.

In human NK cells, CCR7 is present on the CD56^bright^ subset, and alleged responsible for their LN-homing, but absent on the CD56^dim^ subset that dominates in PB (44). Here we could not detect CCR7 expression on bovine NK cells in either PB or AL, or on NK cells from LNs (Lund, unpublished observations) when applying an anti-human CCR7 antibody cross-reactive to bovine cells (45), even though we have previously found moderate expression of mRNA transcripts for CCR7 by bovine NK cells in PB (46) and high expression in LN-resident NK cells (Siddiqui and Hope, unpublished observations). It remains a possibility that post-transcriptional modification of CCR7 halts the surface expression or interferes with antibody binding, and ideally these results should be confirmed with a bovine-specific CCR7 antibody. It was recently shown that bovine γd T cells egress from skin into lymphatic vessels in a CCR7-independent manner (45), whilst the entry of conventional murine and ovine αβ T cells into initial lymphatic vessels relies on CCR7 (47). The inability to detect CCR7 on bovine NK cells suggest that similar to bovine γd T cells, NK cells may migrate in a CCR7-independent manner.

Natural killer cells are responsive to adjuvants (48) and may be important players in vaccine responses (12, 49), underscoring the relevance for harnessing the stimulation of NK cells when designing vaccines. Examining afferent lymphatic NK cells draining the sites of cutaneous vaccination will provide information as to the induction of innate immune cell activation. This could be particularly important in the context of BCG vaccination and infection with *Mycobacteria* where reciprocal interactions between DCs and NK cells lead to enhanced Th1 bias and CD8^+^ T cell activation that has been linked to vaccine efficacy (11, 50). Recent evidence suggests that in humans effective BCG vaccination is dependent upon innate (NK and gamma delta T) cell derived IFN-γ (51, 52), and ongoing studies assessing BCG vaccination and M. bovis infection in cattle have revealed key roles for NK cells [Ref. (53) and unpublished].

The cost and complexity of the cannulation technique, which in the present study was performed at two research sites, resulted in a data material that may contain biasing factors. Steps were taken to limit such confounders: Laboratory analyses were carried out using standardized protocols by the same person, often repeated several times. All animals were recruited from a similar age group since age has proven to be a significant variable in NK cell biology (54–56). Finally, no breed influence was detected in parameters measured in PBMC (Figures 2 and 3). Only one NR dairy calf was successfully catheterized, and since this individual had a high number of NK cells in AL (Figure 1), attention should be paid to a possible breed bias for this parameter. However, its exclusion did not affect statistical or overall conclusions and it was thus included here as an identifiable animal.

In conclusion, we here describe the presence of activated NK cells in AL, suggesting a novel migration route for NK cells from tissues into LNs. LNs may therefore not only be a site for priming of naïve NK cells recruited from PB at the initiation of an immune response (7), but also a site for tissue-activated NK cells arriving via AL that may contribute substantially to the shaping of the adaptive immune response. Further studies of NK cell recirculation under vaccination or infection conditions are needed to fully reveal mechanisms that can be utilized for optimal adjuvant strategies in vaccine development.

## Conflict of Interest Statement

The authors declare that the research was conducted in the absence of any commercial or financial relationships that could be construed as a potential conflict of interest.

## Abbreviations

AL, afferent lymph; FCM, flow cytometry; HEVs, high-endothelial venules; LNs, lymph nodes; MFI, mean fluorescence intensity; PB, peripheral blood; S1PR_1_, sphingosine-1-phosphate receptor 1.
